# Responses of Aroma Related Metabolic Attributes of *Opisthopappus longilobus* Flowers to Environmental Changes

**DOI:** 10.3390/plants12081592

**Published:** 2023-04-10

**Authors:** Zhixia Liu, Yafei Lan, Hao Zhang, Weili Hao, Shan He, Li Liu, Xiaolong Feng, Qiyang Qie, Min Chai, Yiling Wang

**Affiliations:** College of Life Science, Shanxi Normal University, Taiyuan 030031, China

**Keywords:** *Opisthopappus longilobus*, flower aromatics, metabolic pathway

## Abstract

*Opisthopappus longilobus* (*Opisthopappus*) and its descendant species, *Opisthopappus taihangensis*, commonly thrive on the Taihang Mountains of China. Being typical cliff plants, both *O. longilobus* and *O. taihangensis* release unique aromatics. To determine the potential differentiation and environmental response patterns, comparative metabolic analysis was performed on *O. longilobus* wild flower (CLW), *O. longilobus* transplant flower (CLT), and *O*. *taihangensis* wild flower (TH) groups. Significant differences in the metabolic profiles were found, not within *O*. *longilobus*, but between *O*. *longilobus* and *O*. *taihangensis* flowers. Within these metabolites, twenty-eight substances related to the scents were obtained (one alkene, two aldehydes, three esters, eight phenols, three acids, three ketones, three alcohols, and five flavonoids), of which eugenol and chlorogenic were the primary aromatic molecules and enriched in the phenylpropane pathway. Network analysis showed that close relationships occurred among identified aromatic substances. The variation coefficient (CV) of aromatic metabolites in *O*. *longilobus* was lower than *O*. *taihangensis*. The aromatic related compounds were significantly correlated with the lowest temperatures in October and in December of the sampled sites. The results indicated that phenylpropane, particularly eugenol and chlorogenic, played important roles in the responses of *O*. *longilobus* species to environmental changes.

## 1. Introduction

The metabolic processes of organisms are dynamic and complex systems that are regulated by multiple factors [1]. From the most upstream genomic DNA and mRNA, and from proteins to traits, organisms continually adjust their metabolic responses and metabolites to maintain a dynamic balance between their internal and external environments [2]. All reactions, from genes to traits, are the final comprehensive result of the co-regulation of genes and the external environment [3]. Thus, changes in the metabolites of organisms, the relationships between them, and their external manifestations are essential for elucidating their adaptations and evolutionary processes [4]. Conversely, metabolic data can more accurately reflect the physiological status and phenotypic presence of plants while clearly identifying minor changes in genetic and protein expression [2]. 

At different growth stages, differences in the metabolic activities of plants can facilitate the exploration and tracking of changing metabolite distribution patterns [5]. Based on the differences and interactions between metabolites, environmental influences on the metabolites of plants, as well as their adaptive pathways and metabolic networks, may be inferred [6].

Generally, continuous and dynamic changes in the environment typically result in adaptions by plants [7]. These adaptations involve the production and accumulation of a diverse set of metabolites, ranging from signaling hormones and primary metabolites to a wide array of specialized multifunctional metabolites [8,9]. When plants are subject to environmental stress, specialized metabolites can improve their protection and competitiveness for survival [10]. Under different environments, the growth and development of plants can be modified by changing environmental factors [11]. Plants can regulate different signaling pathways by controlling the expression of genes, thus impacting metabolic levels and responding to habitat changes [12]. The synthesis and accumulation of secondary metabolites involve very complex processes that are affected by genetic, developmental, and environmental factors [13]. The secondary metabolism of plant flowers in different habitats were significantly different due to the influences of habitat heterogeneity [14].

In the aspect of the relationships between plant metabolism and the environment, different models have been proposed. Some plants synthesize and accumulate metabolites regardless of their environment. However, for most plants, the synthesis and accumulation of metabolites are altered with changes in the environment [15]. Plants typically allocate various types and quantities of metabolites in response to specific environmental changes. They either synthesize particular metabolites or significantly increase their concentrations under certain environmental conditions [16]. For example, the volatile secondary metabolites of insect repellents in cotton are synthesized after being stimulated by insects, rather than prior to being synthesized and stored [17]. 

During the growth and development of plants, myriad environmental influences can impact the synthesis and accumulation of metabolites, including biological and abiotic factors [17,18]. Further, the effects of environmental factors on plant metabolism are very complex. The synthesis and accumulation of one type of metabolite can be induced by several environmental factors, whereas one type of environmental factor can induce the synthesis and accumulation of multiple metabolites [19]. Identical environmental factors may also have distinct effects on different plants [20]. Although any environmental factor may affect the metabolism of plants for various species under different natural conditions, the degree of influence can vary [20,21]. 

*Opisthopappus longilobus*, belongs to the genus *Opisthopappus*, which is mainly distributed in the Hebei Provinces of China [22]. This species grows on the slopes or cliffs of the Taihang Mountains. It is a typical cliff plant [23] and possesses good cold and drought resistance [24]. *O. longilobus* is rich in aromatic oils with a strong and persistent fragrance and has high ornamental and medicinal value [25,26]. 

Within the *Opisthopappus* genus, *Opisthopappus taihangensis* is another cliff residing species that is primarily located in Henan Province, China. Based on our previous study, *O. taihangensis* is regarded as a descendant of *O. longilobus*. They diverged during the early Miocene under differing precipitation regimes due to the intensification of the Asian monsoon [27]. 

Though the two species present a significant genetic differentiation [22], they exhibit morphological similarities under different habitats [27]. The morphological differentiation occurred only in the leaf segmentation pattern with or without bracts [27,28]. Through field investigations, we observed that the flowers of the two species evolved some morphological differentiation. The inflorescence diameter, tubular flower diameter, ligule length, ligule width, number of ligules, and number of tubular flowers of *O. taihangensis* were larger than those of *O. longilobus*. The petal color of *O. taihangensis* primarily appeared pink and then changed to white, while that of *O. longilobus* occasionally presented as pink at the end of the flowering cycle.

Apart from this, the flowers of both *O. longilobus* and *O. taihangensis* can release unique and potent odors. The aromatic elements of plants generally consist of secondary metabolites. It is uncertain as to whether the metabolites and metabolic pathways of the *O. longilobus* ancestor were identical to those of *O. taihangensis* descendant flowers. If there was any differentiation, what were the key aromatic metabolites involved pathways and associated genes? Under these differentiations, what were the potential mechanisms, and were the adaptive responses to the disruptions caused by different habitats?

To answer the above questions, we initially identified the types of metabolites involved in the flowers of the two species (compared *O. longilobus* wild flowers, *O. longilobus* transplant flowers, and *O. taihangensis* wild flowers). Subsequently, the different aromatic metabolites and metabolic pathways were comparatively analyzed between the *O. longilobus* ancestor and *O. taihangensis* descendant. Finally, the potential mechanisms of *O. longilobus* in response to heterogeneous habitats were elucidated.

## 2. Results

### 2.1. Total Metabolism of the Flowers

In the flower metabolites, a total of 95,655 features were detected in the positive ion mode, and 36,478 features were detected in the negative ion mode. 5439 metabolites were obtained in the positive and negative ion mode, which accounted for 97.6% of those of the flowers. Based on quantitative analysis, 14,262 and 10,004 high-quality substances were obtained in the positive and negative ion modes, respectively. 

**Figure 1:**
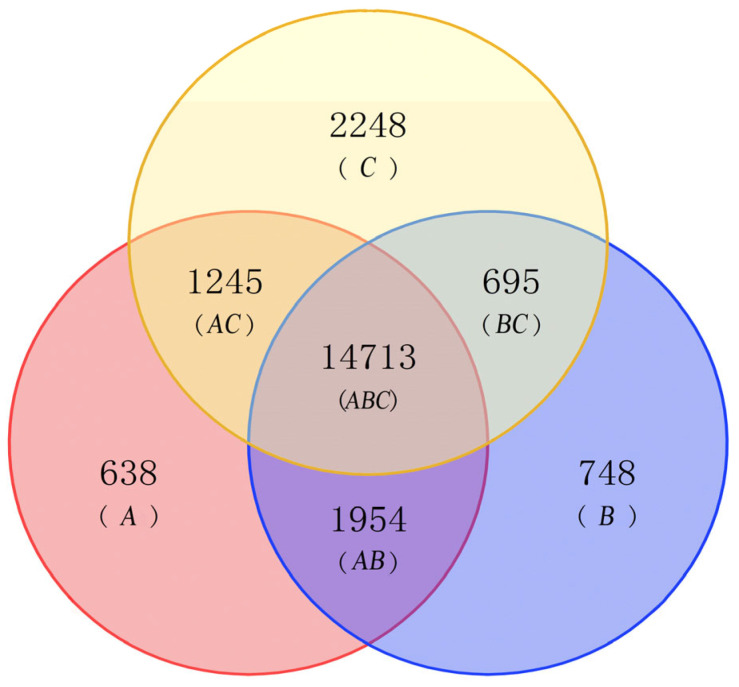
Venn diagrams for flowers metabolites. A. red for CLW; B. blue for CLT; C. yellow for TH. AB. common substances to CLW and CLT; AC. common substances to CLW and TH; BC. common substances to CLT and TH; ABC. common to CLW, CLT, TH groups. Note: CL, *O. longilobus*; CLW, *O. longilobus* wild flowers; CLT, *O. longilobus* transplant flowers; TH, *O. taihangensis* wild flowers.

Among the 3 groups, 14,713 substances were shared through the screened Venn diagram (Figure 1), in which there were 1954 metabolites between CLW and CLT, 1245 between CLW and TH, and 695 between CLT and TH.

There were 638 metabolites that were unique to CLW, 748 to CLT, and 2248 to TH. Significantly distinct metabolic expressions occurred between the CLW, CLT, and TH groups. For the *O. longilobus* wild flowers and *O. longilobus* transplant flowers (CLW/CLT), the differential metabolites were upregulated by 919 and downregulated by 1189. The differential metabolites of wild flowers of *O. taihangensis* and those of *O. longilobus* (TH/CLW) were upregulated by 4020 and downregulated by 3722. Between TH/CLT, the differential metabolites were upregulated by 2560 and downregulated by 3135. 

KEGG enrichment analysis revealed that the differential metabolites were enriched into 47 pathways in CLW/CLT, 59 metabolic pathways in TH/CLW, and 61 pathways in TH/CLT (Table A1). Meanwhile, the significantly different metabolic pathways were all involved in secondary plant metabolite biosynthesis, phenylpropanoid biosynthesis, and arginine biosynthesis between TH/CLT, TH/CLW, and CLW/CLT.

### 2.2. Detection of Aromatic Compounds

Through the screened secondary metabolic data, a total of 28 non-volatile compounds related to aromatic compounds were identified in the *O. longilobus* wildflowers (CLW), *O. longilobus* transplant flowers (CLT), and *O. taihangensis* wild flowers (TH), including one alkene, two aldehydes, three esters, eight phenols, three acids, three ketones, three alcohols, and five flavonoids. These compounds were chlorogenate, caffeate, carvacrol, thymol, eugenol, cinnamaldehyde, scopoletin, coniferyl aldehyde, trans-2-hydroxycinnamate, 4-hydroxycinnamyl aldehyde, 4-hydroxystyrene, quercetin, vitexin, luteolin 7-O-beta-D-glucoside, chrysoeriol, rutin, syringetin, taxifolin, delphinidin, naringenin chalcone, dioctyl phthalate, p-cresol, 3-hydroxybenzaldehyde, phenyl acetate, 3-nitrophenol, trans-2-hydroxycinnamic acid, and hesperetin, respectively. 

Enrichment analysis indicated that 28 identified aromatic compounds were engaged in phenylpropanoid biosynthesis, flavone and flavonol biosynthesis, chemical carcinogenesis-receptor activation, bisphenol degradation, toluene degradation, phenylalanine metabolism, and aminobenzoate degradation.

**Figure 2:**
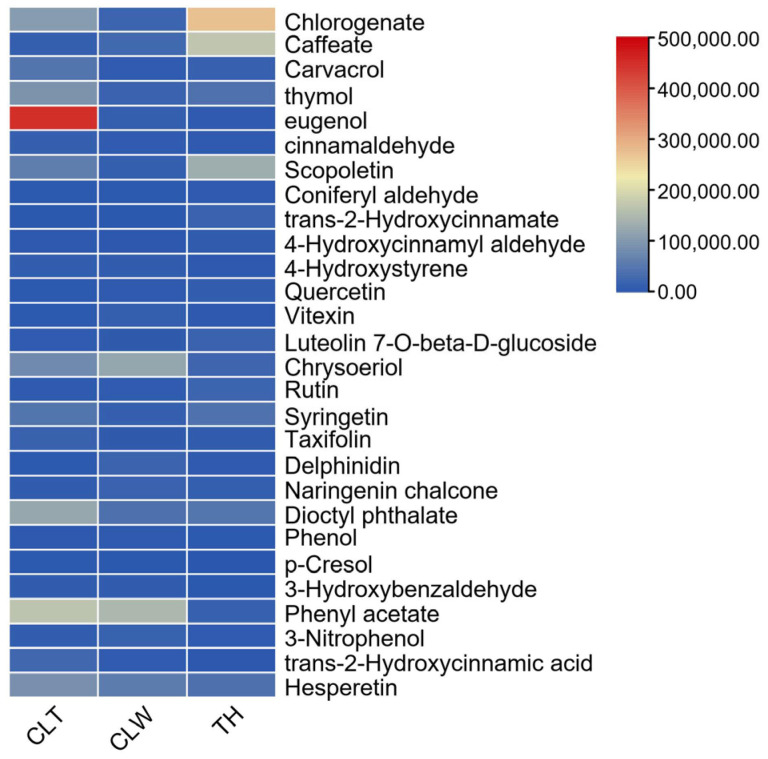
CLT, CLW, and TH group aroma substances expression heatmap. The top right corner is the highest to lowest expression, and red is the highest expression color. Note: The abbreviation of CLT, CLW, and TH was same with Figure 1.

The most highly expressed metabolite for CLT was eugenol (Figure 2 and Table 1), followed by caffeate, carvacrol, thymol, and chlorogenate. The least expressed metabolite was hesperetin.

For CLW, the most highly expressed metabolite was phenyl acetate, followed by chrysoeriol, hesperetin, dioctyl phthalate, and caffeate. The least expressed metabolite was p-Cresol.

**Figure A1:**
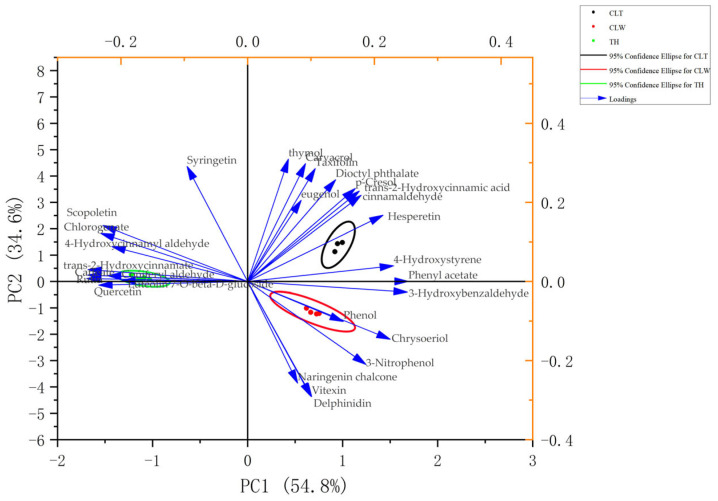
PCA of significant aromatic substances of CLT, CLW, TH groups. Note: The arrows indicate the feature vectors of the aromatic metabolites. The projection of the arrows to the coordinate axis represents the contribution of the original feature to each principal component in the new space. The longer the projection, the greater the contribution of this metabolite to this principal component. Based on the feature vectors of metabolites, it can be clearly seen that the samples from different groups were far apart from each other, while samples from the same group were gathered together. In addition, the black, red, and green dots represent samples from the CLT, CLW, and TH groups, respectively.

In TH, chlorogenate was the most highly expressed metabolite, followed by caffeate, scopoletin, dioctyl phthalate, and syringetin. The least expressed metabolite was p-Cresol. In addition, three groups could be distinguished from each other according to PCA (Figure A1). The overall differences in aromatic substances were obvious among three groups, which indicated that the aromatic compounds in CLT, CLW, and TH were significantly different.

### 2.3. Coefficient of Variation of Aromatic Compounds

Based on the identified aromatic substances, the coefficients of variation (CV) (Table A2) of fragrance metabolism were calculated. For CLT, the CV value ranged from 0.1 to 48.72, whereas for CLW, it ranged from 0.58 to 55.25, and, for TH, this index ranged from 2.17 to 80.90.

Furthermore, chlorogenic and eugenol had the relatively higher CV values among the identified fragrance metabolites.

### 2.4. Network Diagram among Fragrance Metabolites

**Figure 3:**
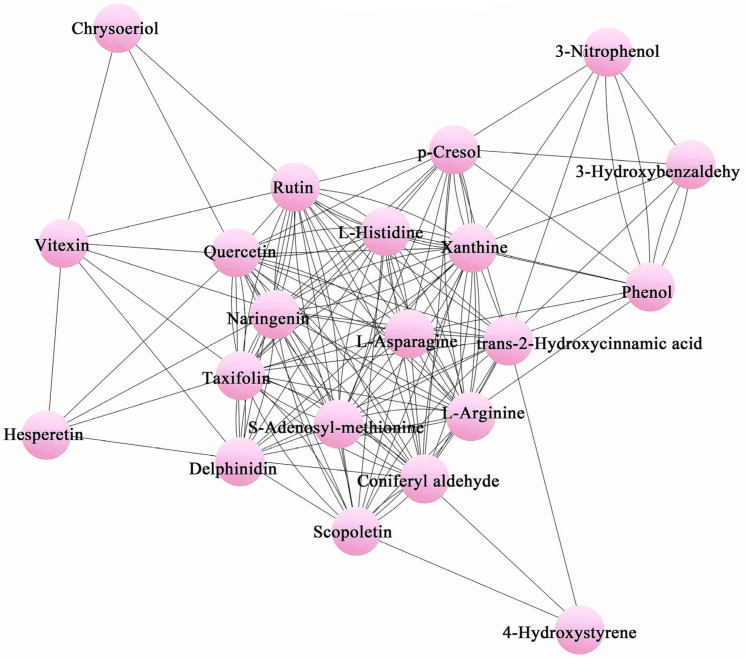
Metabolite network relationship of aromatic substances. Each node represents a metabolite.

A correlation metabolic network was developed for the odor metabolites (Figure 3). Within the network diagram, closely linked metabolites were found likely involved in the same or related metabolic pathways. Most of the metabolites were relatively well connected, which implied that these aromatic substances were fundamental for the growth and survival of *O. longilobus* and *O. taihangensis*.

### 2.5. Environmental Correlation of Aromatic Substances

To explore the environmental correlation of aromatic substances, we firstly investigate the key environmental factors in each sampled site. Then, the correlation analysis was performed between the identified aromatic substances and environmental factors. 

Using the PCA correlation matrix of environmental factors, fourteen variables were primary factors (Table A3). When simultaneously considering the VIF results (Table A3), it was found that two environmental factors, namely the lowest temperatures in October and December, were the most important factors for the sampled sites (Table 2). Thus, the correction analysis was carried out with the two factors.

According to correlation analysis (Table A3 and Table 2), chlorogenic was significantly correlated with the minimum temperature in December, and the Pearson’s correlation coefficient was 0.72105. Eugenol was significantly correlated with the minimum temperatures in October and December, and the Pearson’s correlation coefficients were 0.88483 and 0.82039, respectively.

## 3. Discussion

### 3.1. Different Metabolites of the Flowers

In general, the regulation of primary metabolite allocation can affect fundamental processes such as the growth and development of an organism. In addition, other processes aimed at increasing plant fitness in specific environments are more associated with specialized metabolic processes, such as those involved with protection against herbivores and pathogens, reduction of oxidative stress, or competition with other plants [29]. During various developmental processes, plants need to continuously adjust the distribution of metabolites to maintain growth and survival under changing environments [30]. 

Within the *O. longilobus* species, there were obviously different metabolites between CLW and CLT, for example chlorogenate, eugenol, chrysoeriol, syringetin, and hesperetin. These metabolites mainly enriched the phenylpropanoid biosynthesis, flavone and flavonol biosynthesis, and arginine biosynthesis (Table 1). 

When *O. longilobus* individuals were transplanted from the wild site to the transplant garden, this species adjusted its metabolic pathways, such as phenylpropane biosynthesis, to respond the changed environment to ensure that its flowers bloomed normally.

For plants, arginine biosynthesis pathway was an important one of metabolites pathways. Within arginine biosynthesis pathways, arginine makes a key function in the regulation of abiotic and biotic stress in plants. When plants are exposed to various abiotic stresses, the expression of arginase genes is not only upregulated but also the enzyme activity is increased. When plants are subjected to biotic stresses, such as insect feeding and pathogen infestation, arginase also displays a significant effect. In addition, arginine promoted root growth and improved the salt tolerance of plants [31]. 

For *O. longilobus* and *O. taihangensis*, the significantly enriched arginine biosynthesis pathway might have affected the florescence development and the tolerance to the various stresses. From the evolutionary viewpoint, the *O. taihangensis* descendant would occupy different surroundings compared with its *O. longilobus* ancestor. Through adjusting its metabolites and metabolic pathways, *O. taihangensis* must ensure its development and survival when facing abiotic and biotic stresses from a novel environment. 

### 3.2. Different Aromatic Substances of the Flowers

Plants produce a diversity of secondary metabolites, while generating and storing an extensive range of volatile organic compounds in their flowers. Typically, aromatic substances are important metabolites in plants for attracting insects, resisting diseases and pests, and adapting to altered habitats [32,33]. Further, the scents attract and repel various pollinators for different pollination processes [34,35]. 

**Figure 4:**
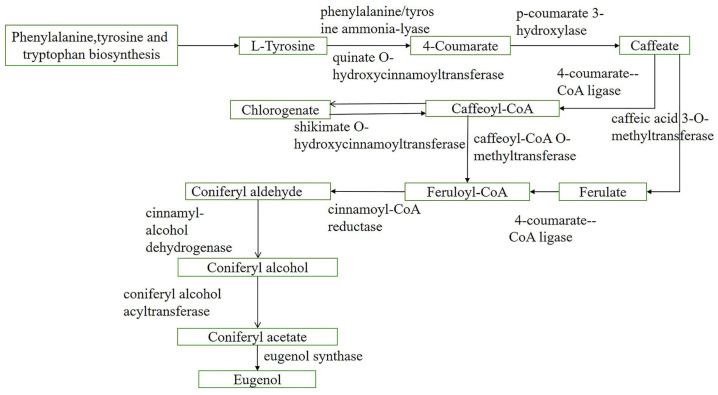
Chlorogenic and eugenol synthetic related pathway in phenylpropanoid biosynthesis pathway.

In the CLW, CLT, and TH groups, the pathways related to flower fragrances were primarily phenylpropanoid, flavone, and flavonol biosynthesis, which are mainly composed of phenylpropanes (such as chlorogenic and eugenol) (Figure 4), which was consistent with the general pattern of the chemical composition of floral scents [36]. 

Metabolism occurs between phenylpropane metabolism and other secondary metabolic pathways, which maintains a dynamic balance between phenylpropane metabolism and resisting environmental degradation [37]. When downregulated, chlorogenic based metabolites can be metabolized to storage forms such as glycosyl based derivatives or decomposed quinic acid and caffeic acid, and can be further metabolized to more complex molecules such as lignin [38]. These contribute to plant development and plant–environment interactions, and phenylpropanoid-based polymers (such as lignin) are required for mechanical support to facilitate growth and long-distance transport of water and nutrients [39]. 

Phenylpropanoid homeostasis between the different branches of phenylpropanoid metabolism exhibits extraordinary complexity and a high-level of plasticity during successive developmental stages in response to environmental stimuli and changes [39,40]. Lignin encapsulates carbohydrates composed of cellulose and hemicellulose to form a composite wood fiber barrier to resist the attack and destruction of plant tissues by microorganisms and the surrounding environment [41]. 

The metabolites of the phenylpropanoid metabolic pathway contribute to the upright growth of the plant, allowing it to better photosynthesize. At the same time, the involved metabolites also effectively protect plants from UV light, diseases, and insects for healthy plant growth. Accordingly, the phenylpropanoid pathway would make *O. longilobus* grow better with the changed habits, ensure its floral development, and maintain its survival and evolution.

Chlorogenic and eugenol are both involved in the phenylpropane pathway. For CLW, CLT, and TH (Figure 4), chlorogenic is upstream and eugenol is downstream. Moreover, the relative substances of synthetic chlorogenic are highly expressed in CLW and CLT (Table 1).

The first step of the chlorogenic metabolic pathway involves the dissociation of ammonia from l-phenylalanine via phenylalanine ammonia lyase and the production of trans-cinnamic acid. This enzyme belongs to the aromatic amino acid lyase family, is the first key enzyme in the phenylpropane pathway [42], and is related to most bioactive metabolites, such as flavonoids and anthocyanins. Meanwhile, the caffeoyl-CoA is the final step in the synthesis of chlorogenic, where 4-coumaryl-CoA ligase is one of the crucial enzymes in the phenylpropane pathway toward the metabolism of other substances, which can catalyze cinnamic acid and its hydroxyl groups. Thus, 4-coumaryl-CoA ligase is of great value in the biosynthesis of phenylpropane compounds such as chlorogenic and flavonoids [43].

Intermediates of the phenylpropane metabolic pathway and their secondary metabolites can not only improve the disease resistance of plants, but also regulate and promote plant resistance to abiotic stresses such as low temperatures, high temperatures, and ultraviolet radiation. This might be an explanation for the difference in floral aromatic substances between two groups (CLW and CLT) of *O*. *longilobus* under different surroundings.

Being the main aromatic compound, chlorogenic plays a key role in the attraction of pollinators and against herbivores and pathogens. Further, chlorogenic is produced via the shikimic acid pathway during aerobic respiration, which exhibits anti-inflammatory, antibacterial, antiviral, and antidepressant pharmacological effects in vitro [44]. In this study, the content of chlorogenic in TH was higher than that of CLT and CLW (Table 1). As the descendant of *O*. *longilobus*, additional chlorogenic might induce *O. taihangensis* to not only attract more and different pollinators to facilitate its reproduction, but also to respond to different environments compared with its ancestor and to better survive and develop. Certainly, higher contents of chlorogenic also may be responsible for the enhanced flower color and relatively different flower size of *O*. *taihangensis* in contrast to *O*. *longilobus*.

On the other hand, eugenol can attract biological pollinators, facilitate seed transmission, and protect against herbivores [45]. It is an important type of phenylpropane volatile that is commonly found in plant flowers and mature fruits, which possesses a variety of biological and therapeutic effects [13]. Eugenol had the highest content in CLT, followed by CLW (Table 1). When *O*. *longilobus* was transplanted into the transplant garden from the wild growth site, eugenol would accumulate to enhance survival under changing environmental conditions. Moreover, eugenol is a semi-volatile phenolic compound derived from plants that has a strong carnation musk odor [46] that creates the different fragrances for *O. longilobus* different from *O. taihangensis*.

To adapt to localized environmental conditions, the plants generate many specialized metabolites that contribute to their health and survival and play roles in their capacities [47]. The emission of fragrances from flowers is regulated by metabolic processes [48]. The biosynthesis and emission of floral fragrances are modulated by the stages of flower maturity, circadian rhythms, and by other environmental factors such as temperature [49,50]. Environmental conditions not only influence the vaporization of volatile compounds from flowers, but also their biosynthesis, particularly under different ambient air temperature regimes, which are known to play major roles in the biosynthesis and release of floral fragrances [50,51].

A significant correlation was found between chlorogenic and the minimum temperature in October, and between eugenol and the minimum temperature in October and December. These two environmental factors might directly affect the expression of chlorogenic and eugenol, or indirectly affect the expression of chlorogenic and eugenol synthase. It was reported that low temperatures can induce the expression of phenylalanine ammonia lyase (PAL) and other phenylpropane metabolism genes [52]. Flower buds could only be formed after low temperature treatment, which may be reasonable for *O. longilobus* and *O. taihangensis* species.

## 4. Materials and Methods

### 4.1. Sample Sites and Materials

The Shennong Mountains inhabit Henan Province, while the Xiangtang Mountains reside in Hebei Province. Both of these mountains occupy branches of the Taihang Mountains Range. Despite the two areas being in the center of the Taihang Mountains, the Xiangtang Mountains are 330 km away from the Shennong Mountains, and the environmental conditions are unique for each. The Shennong Mountains have a warm, temperate continental climate with four distinct seasons. In contrast, the Xiangtang Mountains are home to a warm, temperate, semi-humid, semi-arid continental monsoon climate.

In terms of geographical distribution, the Xiangtang Mountains were a growing area only for *O. longilobus,* while, in the Shennong Mountains, only for *O. taihangensis* was distributed.

Because *O. longilobus* grows on the cliffs of the Taihang Mountains, it is very difficult for our wild investigation. To better observe the growth and development of *O. longiloous*, we established a transplant garden (the *O. longilobus* individuals from the Xiangtang Mountains were transplanted to a garden) in Xiangtangshan National park of China (N36 36°20′–36°34′ N, 114°3′–114°16′ E) in 2016. 

This park is located in Fengfeng Mining District, with an annual dryness of 1.4 degrees. Maximum wind speed in the calendar year is 14 m/s. Spring precipitation accounts for 12.7% of the region’s annual precipitation [53]. The environmental conditions of the park, such as annual average temperature, extreme maximum temperature, and annual average rainfall, are different with the Xiangtang Mountains, though both belong to the same climatic zone. From July to October of 2020, *O. longilobus* flowers were collected, including wild flowers and transplant flowers. Concurrently, *O. taihangensis* wild flowers were collected as comparative samples from the Shennong Mountains due to the phylogenetic relationship with *O. longilobus* [54] (Table A4).

For sampling, fresh and healthy whole flowers in the full bloom stage were collected and placed into Ziploc bags (one bag for each group). The samples were quickly fixed with liquid nitrogen and stored at −80 °C. All flower samples were processed as three groups: *O. longilobus* wild flowers (four samples), *O. longilobus* transplant flowers (three samples), and *O. taihangensis* wild flowers (five samples). We remarked the *O. longilobus* wild flowers to CLW, *O. longilobus* transplant flowers to CLT, and *O. taihangensis* wild flowers to TH. CL and TH came from the name of the Chinese phonetic transcriptions of *O. longilobus* and *O. taihangensis*, respectively. Wild was abbreviated as W, while transplant was T. For *O. taihangensis*, the only flowers from the wild were marked as TH.

Meanwhile, our study was conducted in accordance with the laws of the People’s Republic of China, and field collection was approved by the Chinese Government. All researchers received permission letters from the School of Life Science, Shanxi Normal University. The voucher specimens were deposited in the herbarium of School of Life Science, Shanxi Normal University (No: 20200105030–20200105042).

### 4.2. Total Metabolite of the Flowers

#### 4.2.1. Extraction of Total Metabolite

Due to the robustness, high selectivity and sensitivity, and a strong potential for both quantification and identification purposes, the liquid chromatography coupled to tandem mass spectrometry (LC-MS/MS) was performed in this study [55,56,57,58,59]. 

The collected samples were thawed on ice, 100 mg of tissue was weighed and grinded with liquid nitrogen, 120 µL of a precooled 50% methanol buffer was added, and the metabolites were extracted from 20 µL of each sample. 

Subsequently, the metabolite mixture was vortexed for 1 min and incubated for 10 min at room temperature and stored at −20°C overnight. The mixture was then centrifuged at 4000× *g* for 20 min, and the supernatant was transferred to 96-well plates and stored at −80°C pending LC-MS analysis. Pooled quality control (QC) samples were also prepared by combining 10 μL of each extraction mixture. 

All samples were analyzed using a TripleTOF 5600 Plus highresolution tandem mass spectrometer (SCIEX, Warrington, UK) with both positive and negative ion modes. Chromatographic separation was performed using an ultraperformance liquid chromatography (UPLC) system (SCIEX, UK). An ACQUITY UPLC T3 column (100 mm*2.1 mm, 1.8 µm, Waters, UK) was used for the reversed-phase separation. It was introduced for the separation of metabolites, and the mobile phase consisted of solvent A (water, 0.1% formic acid) and solvent B (Acetonitrile, 0.1% formic acid). The gradient elution conditions were as follows: with a flow rate of 0.4 mL/min: 5% solvent B for 0–0.5 min; 5–100% solvent B for 0.5–7 min; 100% solvent B for 7–8 min; 100–5% solvent B for 8–8.1 min; and 5% solvent B for 8.1–10 min. The column temperature was maintained at 35 °C. 

The TripleTOF 5600 Plus system was used to detect metabolites eluted from the column. The curtain gas pressure was set at 30 PSI, the ion source gas1 and gas2 pressure was set at 60 PSI. The interface heater temperature was 650 °C. For the positive-ion mode, the ion spray floating voltage was set at 5 kV, and for the negative-ion mode, it was set at −4.5 kV. The MS data were acquired in the IDA mode. The TOF mass range was 60–1200 Da. Survey scans were acquired every 150 ms, and as many as 12 product ion scans were collected if the threshold of 100 counts/s was exceeded with a 1+ charge state. The total cycle time was fixed at 0.56 s. Four-time bins were summed for each scan at a pulse frequency of 11 kHz by monitoring the 40 GHz multichannel TDC detector with four-anode/channel detection. Dynamic exclusion was set for 4 s. 

#### 4.2.2. Determination of Differential Metabolites

The pretreatment of acquired LC-MS data was performed using XCMS software. Raw data files were converted to mzXML format and then processed using the XCMS, CAMERA, and metaX toolbox included in R software. The data was matched to in-house and public databases. The peak intensity data was further preprocessed using metaX, and metabolic substances with standard deviations of >30% were removed. The dataset groups were normalized prior to analysis using the probabilistic quotient normalization algorithm. The *p* value analyzed by Student’s *t*-test was adjusted for multiple tests using an FDR (Benjamini–Hochberg), and then used for the selection of various metabolites. In conjunction with multivariate statistical analysis, the VIP value obtained by PLS-DA was analyzed to screen differential metabolic ions. The different ions met the following requirements: (1) Ratio ≥ 2 or Ratio ≤ 1/2; (2) Q value ≤ 0.05; (3) VIP ≥ 1 for the screening of differential metabolites. Assessment of nutritional value and quantitative analysis of bioactive phytochemicals was performed through the targeted LC-MS/MS method.

For annotation of differential metabolites, the metabolomic database was annotated using KEGG to retrieve all the pathways mapped by the differential metabolites. The pathways of the differential metabolites were further screened to identify key pathways that had the highest correlations with the different metabolites. Fold-change analysis and the *t*-test statistical test were employed to conduct BH corrections to obtain the Q-value.

#### 4.2.3. Venn Diagram among Differential Metabolites

Subsequently, enrichment analysis of differential metabolites among the CLW, CLT, and TH groups was also performed. In addition, those with different expressions were screened by Origin to develop a Venn diagram. 

### 4.3. Aromatic Substances of the Flowers

#### 4.3.1. Determination of Aromatic Substances

To evaluate the biochemical differences between *O. longilobus* and *O*. *taihangensis* flowers under various conditions, the metabolic scent profiles were compared. According to the original data, the differential metabolites of *p* < 0.05 and VIP > 1 were screened, and the similarity >700 was also screened. The non-volatile aromatic-related metabolites were then identified and classified, and a metabolic pathway map was illustrated using Visio software (Office Visio 2013, Microsoft USA, Redmond, WA, USA).

#### 4.3.2. Variation Coefficient of Identified Aromatic Compounds

The variation coefficient (CV) is a statistic index used to quantify the degree of variation for each metabolite. For this study, the variation coefficient was calculated using the formula: CV = σ/μ, where σ is the standard deviation and μ is the average value. The variation coefficients of different metabolic aromatic compounds were subsequently plotted using SPSS (IBM SPSS Statistics 26, Chicago, IL, USA) software.

#### 4.3.3. PCA (Principal Component Analysis) and Heat Map of Different Aromatic Compounds

A PCA diagram of different aromatic metabolites was plotted using Origin (Origin 2018) software. The relative content maps of differential metabolites were plotted with SigmaPlot version 10.0 (Systat Software Inc., San Jose, CA, USA) software. The metabolic pathway map was depicted using Visio software (Office Visio 2013, Microsoft USA).

#### 4.3.4. Network Relationship among Different Aromatic Metabolites

To further explore the relationship among differential aromatic metabolites, the network analysis was displayed using Cytoscape 3.7.0 software.

### 4.4. Correlations between Floral Metabolites and Environment Factors

The required environmental factors were mainly obtained from the FAO (http://www.fao.org/home/zh/) (accessed on 10 May 2021) and Worldclim (https://worldclim.org/) (accessed on 10 May 2021) websites. Data on the climate of the Taihang Mountains (1970–2000) were mainly obtained from WorldClim 2.1 (www.worldclim.org) (accessed on 20 May 2021), and land data were obtained from FAO (www.fao.org) (accessed on 20 May 2021). 

A total of 103 climatic factors were obtained according to their longitude and latitude using R software. According to the variance inflation factor (VIF) and PCA of Origin software, significant environmental factors were obtained. 

Finally, Pearson correlations between significant environmental factors and different aromatic substances were then calculated and plotted using Origin (Origin 2018) software [60]. It was generally believed that there was a significant relationship when the *p* value was < 0.05.

## 5. Conclusions

Through LC-MS/MS methods, the expressions of metabolic compounds were shown to be significantly different between *O*. *longilobus* wild flowers (CLW), *O*. *longilobus* l transplant flowers (CLT), and *O. taihangensis* wild flowers (TH). Two types of phenylpropane metabolites, eugenol, and chlorogenic, were responsible for the differences in non-volatile aromatic components between *O*. *longilobus* and *O. taihangensis*. Following transplantation, *O. longilobus* exhibited a positive response toward adapting to biological and abiotic stresses via the phenylpropanoid biosynthesis pathway compared with the wild *O. longilobus* flowers. Different aromatic substances were also observed between the two species, which indicated the differences in attracting pollinators, resisting diseases and pests, and adapting to habitats, which may be the reason that the descendant *O. taihangensis* could colonize the completely different environment compared with its ancestor *O. longilobus*. The results in this study revealed the potential response mechanisms of *O. longilobus* to different habitats, while providing a scientific basis for the synthesis and regulation of aromatic substances in *O. longilobus* and *O. taihangensis.*

## Figures and Tables

**Table 1:** Aromatic compound expression (relative units) in CLT, CLW, and TH.

Substance	CLT	CLW	TH	Pathway
Chlorogenate	105,479.21	19,921.39	274,799.37	Phenylpropanoid biosynthesis
Caffeate	10,786.84	26,437.30	169,303.97	Phenylpropanoid biosynthesis
Carvacrol	45,265.46	5310.48	13,994.04	Phenylpropanoid biosynthesis
Thymol	92,435.68	16,687.11	40,157.56	Phenylpropanoid biosynthesis
Eugenol	450,751.55	11,388.27	3814.11	Phenylpropanoid biosynthesis
Cinnamaldehyde	12,952.79	5892.94	4449.50	Phenylpropanoid biosynthesis
Scopoletin	59,001.51	10,522.40	132,320.74	Flavone and flavonol biosynthesis
Coniferyl aldehyde	2351.69	2560.67	4449.50	Phenylpropanoid biosynthesis
Trans-2-HyDdroxycinnamate	1242.84	1394.90	16,694.42	Phenylpropanoid biosynthesis
4-Hydroxycinnamyl aldehyde	3320.20	1792.73	7750.14	Phenylpropanoid biosynthesis
4-Hydroxystyrene	9294.67	7470.99	2751.54	Phenylpropanoid biosynthesis
Quercetin	2609.50	3928.13	9140.53	Flavone and flavonol biosynthesis
Vitexin	2274.42	9813.69	3345.64	Flavone and flavonol biosynthesis
Luteolin 7-O-beta-D-glucoside	6273.09	7178.23	16,711.09	Flavone and flavonol biosynthesis
Chrysoeriol	78,813.62	120,830.08	21,661.31	Flavone and flavonol biosynthesis
Rutin	4227.24	5222.34	20,086.61	Flavone and flavonol biosynthesis
Syringetin	45,869.14	12,767.33	41,409.86	Flavone and flavonol biosynthesis
Taxifolin	15,370.62	6753.96	8319.35	Flavone and flavonol biosynthesis
Delphinidin	2436.45	18,204.98	4483.08	arginine biosynthesis
Naringenin chalcone	8136.07	16,785.24	10,369.81	Flavone and flavonol biosynthesis
Dioctyl phthalate	123,258.45	39,622.58	47,210.77	Chemical carcinogenesis—Receptor activation
Phenol	3296.28	3956.37	2215.23	Bisphenol degradation
p-Cresol	1951.54	514.12	327.25	Toluene degradation
3-Hydroxybenzaldehyde	8049.15	7936.81	1037.18	Toluene degradation
Phenyl acetate	166,712.44	148,542.13	13,535.54	Phenylpropanoid biosynthesis
3-Nitrophenol	8723.93	14,561.86	5941.29	Aminobenzoate degradation
Trans-2-Hydroxycinnamic acid	25,270.25	4991.71	1565.01	Phenylpropanoid biosynthesis
Hesperetin	88,158.46	56,960.99	39,691.62	Flavone and flavonol biosynthesis

Note: the abbreviation of CLT, CLW, and TH was same Figure 1.

**Table 2:** Correlations between aromatic substances and two environmental factors.

	Lowest Temperature in October	Lowest Temperature in October	Lowest Temperature in December	Lowest Temperature in December
	Pearson correlation	*p*-value	Pearson correlation	*p*-value
Chlorogenate	−0.2601	0.41424	0.72105 *	0.00814
Caffeate	−0.54904	0.06449	0.47613	0.11764
Carvacrol	0.93519 *	8.07 × 10^−6^	0.73249 *	0.00674
thymol	0.88483 *	1.31 × 10^−4^	0.82039 *	0.00108
eugenol	0.88483 *	1.31 × 10^−4^	0.82039 *	0.00108
cinnamaldehyd	0.95583 *	1.23 × 10^−6^	0.3087	0.32893
Scopoletin	−0.21318	0.50589	0.75863 *	0.00423
Coniferyl aldehyde	−0.44013	0.15218	0.38082	0.22198
trans-2-Hydroxycinnamate	−0.49241	0.10389	0.52838	0.0774
4-Hydroxycinnamyl aldehyde	−0.28309	0.37261	0.60134 *	0.03861
4-Hydroxystyrene	0.60689 *	0.03639	−0.30418	0.33643
Quercetin	−0.55199	0.06278	0.35871	0.2522
Vitexin	−0.37871	0.22477	−0.98711	2.75 × 10^−9^
Luteolin 7-O-beta-D-glucoside	−0.42553	0.16785	0.38042 *	0.22251
Chrysoeriol	0.19279	0.54829	−0.77187 *	0.00327
Rutin	−0.5253	0.07946	0.49815	0.0993
Syringetin	0.36739	0.24007	0.97295 *	1.09 × 10^−7^
Taxifolin	0.9309 *	1.10 × 10^−5^	0.68244 *	0.01447
Delphinidin	−0.36309	0.24603	−0.9719 *	1.32 × 10^−7^
Naringenin chalcone	−0.39108	0.20873	−0.80275 *	0.00167
Dioctyl phthalate	0.86946 *	2.39 × 10^−4^	0.53605	0.07241
Phenol	0.14631	0.65002	−0.48033	0.11398
p-Cresol	0.97346 *	9.92 × 10^−8^	0.37682	0.22728
3-Hydroxybenzaldehyde	0.49574	0.10121	−0.52455	0.07997
Phenyl acetate	0.56086	0.05782	−0.46192	0.13059
3-Nitrophenol	−0.0152	0.9626	−0.87167 *	2.20 × 10^−4^
trans-2-Hydroxycinnamic acid	0.98848 *	1.57 × 10^−9^	0.34875	0.26656
Hesperetin	0.89367 *	8.93 × 10^−5^	0.07483	0.81721

Note: * indicated a significant relationship occurred.

## Data Availability

All data generated or analyzed during this study are included in this published article.

## Appendix A

**Table A1:** KEGG metabolic pathway enrichment results of the differential metabolites of CLW/CLT, TH/CLW, and TH/CLT flowers.

	TH/CLT		TH/CLW		CLW/CLT	
	Pathway	*p* Value	Pathway	*p* Value	Pathway	*p* Value
1	Aminoacyl–tRNA biosynthesis	0.0000001	Flavone and flavonol biosynthesis	0.000001	Phenylpropanoid biosynthesis	0.000126
2	Biosynthesis of plant secondary metabolites	0.0000018	Aminoacyl–tRNA biosynthesis	0.000002	Flavonoid biosynthesis	0.000146
3	Purine metabolism	0.0000134	Purine metabolism	0.000003	Biosynthesis of plant secondary metabolites	0.000603
4	Arginine biosynthesis	0.0000260	Phenylpropanoid biosynthesis	0.000014	Arginine biosynthesis	0.000669
5	Biosynthesis of amino acids	0.0000294	Biosynthesis of amino acids	0.000051	D-Arginine and D-ornithine metabolism	0.002512
6	Alanine, aspartate, and glutamate metabolism	0.0000715	Alanine, aspartate, and glutamate metabolism	0.000096	Monobactam biosynthesis	0.003177
7	Phenylpropanoid biosynthesis	0.0000787	Biosynthesis of plant secondary metabolites	0.000125	Plant hormone signal transduction	0.003649
8	Flavone and flavonol biosynthesis	0.0001179	Arginine biosynthesis	0.000566	alpha-Linolenic acid metabolism	0.003928
9	ABC transporters	0.0001642	Glutathione metabolism	0.003880	Purine metabolism	0.005093
10	Glycosylphosphatidylinositol (GPI)–anchor biosynthesis	0.0007732	Nitrogen metabolism	0.003933	Ascorbate and aldarate metabolism	0.005406
11	Nitrogen metabolism	0.0033012	alpha-Linolenic acid metabolism	0.005587	Aminoacyl-tRNA biosynthesis	0.007179
12	Monobactam biosynthesis	0.0034142	Flavonoid biosynthesis	0.005988	Glycerophospholipid metabolism	0.007179
13	Biosynthesis of plant hormones	0.0045998	Biosynthesis of plant hormones	0.005988	Nicotinate and nicotinamide metabolism	0.008390
14	Phenylalanine metabolism	0.0058746	Arginine and proline metabolism	0.009545	Biosynthesis of secondary metabolites	0.008622
15	Histidine metabolism	0.0067199	Glycerophospholipid metabolism	0.011879	Caffeine metabolism	0.011118
16	Arginine and proline metabolism	0.0073812	Pantothenate and CoA biosynthesis	0.011902	Valine, leucine, and isoleucine biosynthesis	0.013267
17	Glycerophospholipid metabolism	0.0096015	Nicotinate and nicotinamide metabolism	0.014398	Carbon fixation in photosynthetic organisms	0.013267
18	D-Arginine and D-ornithine metabolism	0.0107750	Sulfur relay system	0.014663	Biosynthesis of plant hormones	0.014980
19	Sulfur relay system	0.0130323	Carbapenem biosynthesis	0.017159	ABC transporters	0.015198
20	beta-Alanine metabolism	0.0145440	beta-Alanine metabolism	0.017159	Stilbenoid, diarylheptanoid, and gingerol biosynthesis	0.015581
21	Plant hormone signal transduction	0.0154762	Plant hormone signal transduction	0.017401	Ether lipid metabolism	0.015581
22	Biosynthesis of secondary metabolites	0.0155926	Biosynthesis of secondary metabolites	0.019561	Biosynthesis of amino acids	0.015608
23	Cysteine and methionine metabolism	0.0175387	ABC transporters	0.020756	Phenylalanine metabolism	0.017460
24	Isoflavonoid biosynthesis	0.0185040	Cysteine and methionine metabolism	0.021527	Cutin, suberine, and wax biosynthesis	0.018056
25	Lysine biosynthesis	0.0185420	Lysine biosynthesis	0.021826	Alanine, aspartate, and glutamate metabolism	0.019352
26	Phenylalanine, tyrosine, and tryptophan biosynthesis	0.0185420	Phenylalanine, tyrosine, and tryptophan biosynthesis	0.021826	Linoleic acid metabolism	0.019352
27	Glutathione metabolism	0.0230920	2-Oxocarboxylic acid metabolism	0.027176	Pantothenate and CoA biosynthesis	0.019352
28	Flavonoid biosynthesis	0.0238246	Monobactam biosynthesis	0.029026	Pyruvate metabolism	0.023468
29	alpha-Linolenic acid metabolism	0.0300204	Zeatin biosynthesis	0.029026	beta-Alanine metabolism	0.024913
30	Cyanoamino acid metabolism	0.0358597	Phenylalanine metabolism	0.034937	Lysine biosynthesis	0.029461
31	Ascorbate and aldarate metabolism	0.0400552	Cyanoamino acid metabolism	0.041900	Cyanoamino acid metabolism	0.046743
32	Caffeine metabolism	0.0448472	Histidine metabolism	0.046734	Histidine metabolism	0.050557
33	Glycine, serine, and threonine metabolism	0.0467952	Caffeine metabolism	0.050120	Flavone and flavonol biosynthesis	0.054482
34	Taurine and hypotaurine metabolism	0.0488251	Glycine, serine, and threonine metabolism	0.054479	Glycine, serine, and threonine metabolism	0.056484
35	Carbon fixation in photosynthetic organisms	0.0529265	Taurine and hypotaurine metabolism	0.054530	Fatty acid biosynthesis	0.056484
36	Nicotinate and nicotinamide metabolism	0.0591885	Valine, leucine, and isoleucine biosynthesis	0.059071	Biosynthesis of unsaturated fatty acids	0.064746
37	Stilbenoid, diarylheptanoid, and gingerol biosynthesis	0.0614800	Carbon fixation in photosynthetic organisms	0.059071	Pentose and glucuronate interconversions	0.066873
38	Ether lipid metabolism	0.0614800	Stilbenoid, diarylheptanoid, and gingerol biosynthesis	0.068528	Cysteine and methionine metabolism	0.082391
39	2-Oxocarboxylic acid metabolism	0.0653205	Ether lipid metabolism	0.068528	2-Oxocarboxylic acid metabolism	0.083403
40	Linoleic acid metabolism	0.0751166	Linoleic acid metabolism	0.083564	Isoflavonoid biosynthesis	0.084693
41	Biotin metabolism	0.0751166	Biotin metabolism	0.083564	Anthocyanin biosynthesis	0.091717
42	Pantothenate and CoA biosynthesis	0.0751166	Glyoxylate and dicarboxylate metabolism	0.087651	Arginine and proline metabolism	0.116284
43	Glyoxylate and dicarboxylate metabolism	0.0758846	Isoflavonoid biosynthesis	0.094429	Sesquiterpenoid and triterpenoid biosynthesis	0.147696
44	Metabolic pathways	0.0936330	Pyruvate metabolism	0.099500	Amino sugar and nucleotide sugar metabolism	0.203258
45	Carbapenem biosynthesis	0.0946195	C5-Branched dibasic acid metabolism	0.104990	Carbon metabolism	0.214688
46	C5-Branched dibasic acid metabolism	0.0946195	Tyrosine metabolism	0.151417	Metabolic pathways	0.426795
47	Glycerolipid metabolism	0.1100949	Butanoate metabolism	0.157551	beta-Alanine metabolism	0.150846
48	Zeatin biosynthesis	0.1316694	Galactose metabolism	0.182275		
49	Tyrosine metabolism	0.1326520	Lysine degradation	0.226724		
50	Butanoate metabolism	0.1427985	Carbon metabolism	0.305920		
51	Lysine degradation	0.2068783	Anthocyanin biosynthesis	0.317058		
52	Fructose and mannose metabolism	0.2188817	Tropane, piperidine, and pyridine alkaloid biosynthesis	0.329871		
53	Pentose and glucuronate interconversions	0.2249079	Pyrimidine metabolism	0.329871		
54	Carbon metabolism	0.2740372	Metabolic pathways	0.360415		
55	Tropane, piperidine, and pyridine alkaloid biosynthesis	0.3038096	Glucosinolate biosynthesis	0.374164		
56	Pyrimidine metabolism	0.3038096	Porphyrin and chlorophyll metabolism	0.410889		
57	Glucosinolate biosynthesis	0.3459471	Sesquiterpenoid and triterpenoid biosynthesis	0.453081		
58	Porphyrin and chlorophyll metabolism	0.3729929	Ubiquinone and other terpenoid–quinone biosynthesis	0.464742		
59	Ubiquinone and other terpenoid–quinone biosynthesis	0.4331321	Diterpenoid biosynthesis	0.536900		
60	Diterpenoid biosynthesis	0.5036407				
61	Amino sugar and nucleotide sugar metabolism	0.5292665				

**Table A2:** Coefficient of variation (CV) for CLT, CLW, and TH.

Substance	CLT-CV	CLW-CV	TH-CV
Chlorogenate	4.36	2.59	8.80
Caffeate	3.97	3.03	4.60
Carvacrol	11.79	20.25	6.01
Thymol	7.58	6.66	3.03
Eugenol	6.54	5.70	4.05
Cinnamaldehyd	4.25	24.60	6.88
Scopoletin	3.61	8.01	4.96
Coniferyl aldehyde	11.83	55.25	6.88
Trans-2-Hydroxycinnamate	48.72	29.11	8.26
4-Hydroxycinnamyl aldehyde	3.16	23.66	29.63
4-Hydroxystyrene	8.34	34.21	2.17
Quercetin	47.03	33.96	20.87
Vitexin	17.04	6.76	9.71
Luteolin	1.46	53.42	31.48
Chrysoeriol	5.49	4.31	14.87
Rutin	10.91	7.93	5.42
Syringetin	4.28	4.80	10.89
Taxifolin	7.36	9.03	10.61
Delphinidin	13.97	10.22	38.06
Naringenin chalcone	3.00	6.24	36.66
Dioctyl phthalate	21.35	39.00	38.26
Phenol	35.77	44.32	10.05
p-Cresol	18.11	5.94	33.29
3-Hydroxybenzaldehyde	6.76	9.14	27.61
Phenyl acetate	7.71	6.63	10.97
3-Nitrophenol	1.65	9.08	10.75
trans-2-Hydroxycinnamic acid	8.11	4.38	14.84
Hesperetin	3.78	7.47	11.98
Rrange	0.1–48.72	0.58–55.25	2.17–38.06

**Table A3:** Major environmental factors of sample sites.

Environmental Factors	PCA	VIF
The coldest month minimum temperature	0.23253	-
Lowest temperature January	0.23253	-
Lowest temperature December	0.23059	1.502
Average temperature December	0.21505	-
Water vapor pressure Feb	0.20949	-
Lowest temperature November	0.19197	-
Water vapor pressure April	0.18347	-
Average precipitation November	0.18101	-
Average temperature January	0.17675	-
Lowest temperature February	0.16969	-
Driest quarterly average temperature	0.1695	-
Coldest quarterly average temperature	0.1695	-
Water vapor pressure March	0.16804	-
Lowest temperature October	0.15701	1.460

**Table A4:** CLT, CLW, and TH samples and location information.

Species	Sample	Location	Batch	Class
*Opisthopappus longilobus*	CLW1	Xiangtang Moutains, Hebei	1	CLW
	CLW2	Xiangtang Moutains, Hebei	1	CLW
	CLW3	Xiangtang Moutains, Hebei	1	CLW
	CLW4	Xiangtang Moutains, Hebei	1	CLW
	CLT1	Xiangtangshan National Park, Hebei	1	CLT
	CLT2	Xiangtangshan National Park, Hebei	1	CLT
	CLT3	Xiangtangshan National Park, Hebei	1	CLT
*Opisthopappus taihangensis*	TH1	Shennong Moutains, Henan	1	TH
	TH2	Shennong Moutains, Henan	1	TH
	TH3	Shennong Moutains, Henan	1	TH
	TH4	Shennong Moutains, Henan	1	TH
	TH5	Shennong Moutains, Henan	1	TH
